# Reassessing the Role of Root Hairs in Adaptation to Low Phosphorus Conditions: Lessons From Wild Barley

**DOI:** 10.1111/ppl.71062

**Published:** 2026-08-11

**Authors:** Tahmina Nazish, Yunpeng Tao, Jiayin Pang, Meixue Zhou, Fanrong Zeng, Zhong‐Hua Chen, Sergey Shabala

**Affiliations:** ^1^ School of Biological Sciences The University of Western Australia Perth Western Australia Australia; ^2^ The UWA Institute of Agriculture The University of Western Australia Perth Western Australia Australia; ^3^ Tasmania Institute of Agriculture University of Tasmania Hobart Tasmania Australia; ^4^ MARA Key Laboratory of Sustainable Crop Production in the Middle Reaches of the Yangtze River, College of Agriculture Yangtze University Jingzhou China; ^5^ School of Agriculture, Food and Wine, Waite Research Institute University of Adelaide Glen Osmond South Australia Australia; ^6^ International Research Centre for Environmental Membrane Biology Foshan University Foshan Guangdong China

**Keywords:** *Hordeum spontaneum*, *Hordeum vulgare*, low‐phosphorus tolerance, phosphorus acquisition efficiency, root hair plasticity

## Abstract

Phosphorus (P) deficiency is one of the most widespread constraints on global crop productivity. Root hairs are widely regarded as a pivotal tissue for P acquisition, yet their quantitative contribution to P uptake and subsequent plant performance remains unclear. We examined 12 cultivated and 12 wild barley genotypes, classified as tolerant or sensitive to low P based on relative total biomass, and quantified root hair number and length in relation to plant performance. While P deficiency generally stimulated root hair proliferation, contrasting strategies emerged: tolerant genotypes have maintained growth and P status with minimal changes in root hair traits, whereas sensitive genotypes depended strongly on root hair proliferation and elongation, which correlated positively with root and shoot biomass as well as shoot P status. In tolerant genotypes, negative correlations between shoot performance and root hair traits indicated that enhanced root hair development often represents a compensatory response or even a trade‐off, where carbon allocation to epidermal traits does not yield proportional benefits. These results suggest that metabolically inexpensive traits aid stress adaptation in sensitive backgrounds. Crucially, root hair traits are not a reliable proxy for P efficiency, as their functional value varies across genetic backgrounds. Our findings challenge the prevailing view that longer or denser root hairs universally improve P uptake and call for a reappraisal of how visible morphological responses are interpreted. These insights emphasize that breeders should integrate root hair plasticity into broader root system ideotypes, rather than targeting it in isolation, to enhance P efficiency in cereal crops.

## 1 Introduction

Phosphorus (P) is a vital macronutrient crucial for plant growth and development as it constitutes numerous important biomolecules and takes part in essential physiological processes, such as photosynthesis and respiration (Lambers 2022). Plants take up P as inorganic phosphate (Pi), but its poor mobility and high fixation in soil result in its low biological availability (Han et al. 2022), with a major negative impact on crop productivity. The modern agriculture relies heavily on the usage of P fertilizers (Chen et al. 2016); however, a significant proportion of these fertilizers is not absorbed by plants but is fixed or leached away, resulting in significant environmental degradation caused by eutrophication (Chtouki et al. 2024). Moreover, the source of P fertilizers (rock phosphate) is in finite supply (Dawson and Hilton 2011), necessitating the need to unravel the traits that can enhance P acquisition in plants, particularly under low‐P conditions.

Phosphorus uptake by plants often generates a depletion zone around the roots owing to its low concentration and restricted movement in the soil (Poirier et al. 2022). Plants cope with P shortage via several mechanisms; one of them is the modification of root system architecture for enhanced P uptake (Lu et al. 2024). Phosphorus deficiency leads to the suppression of primary root elongation, stimulation of lateral root formation, and a reduction in gravitropic response, collectively producing a denser root network concentrated in the upper layers of the soil (Lu et al. 2024). This is complemented by upregulated high‐affinity Pi transporters, secretion of P‐mobilizing compounds, and recruitment of beneficial microbes under P‐deficient conditions (Yang et al. 2024).

Another notable modification in plant roots under low‐P environment is a boost in the number and length of root hairs (Ibeas et al. 2024). Root hairs are temporary, tube‐like extensions of root epidermal cells acting as a hub for numerous nutrient transporters and extend the root surface area in contact with soil, helping plants to acquire P efficiently from a larger volume of soil (Bates and Lynch 2001; Kochian et al. 2015). It has been demonstrated that longer and persistent root hairs provide an optimal cost–benefit trade‐off, enhancing P‐uptake efficiency even under low‐P conditions while keeping the metabolic expenditure for root development and maintenance at a manageable level (Brown et al. 2013). Species with longer and denser root hairs exhibit enhanced P uptake by increasing root surface area for nutrient acquisition (Ma et al. 2001; Miguel et al. 2015). Moreover, considerable variation in root hair length and density exists among species or different genotypes within a single species, reflecting underlying genetic diversity and adaptive responses to nutrient stress. In barley ( *Hordeum vulgare* L.), for example, the presence of root hairs conferred an advantage in root hair‐bearing genotypes performing better than hairless counterparts in both soil penetration and P uptake efficiency (Haling et al. 2013). While the role of root hairs in P uptake is well acknowledged, their precise contribution under low‐P stress has yet to be clearly defined in several crops such as barley.

Domesticated from its hulled type, two‐rowed, and brittle‐rachised wild progenitor ( *Hordeum vulgare ssp. spontaneum* ) as early as 12,000 years ago in the Fertile Crescent (Azhaguvel and Komatsuda 2007; Zohary et al. 2012), barley is one of the earliest cultivated crops in human history and has become widespread globally as livestock feed and a mainstay in brewing (Jiang et al. 2025). Moreover, it is the principal cereal crop cultivated at extreme altitudes, reaching up to 4700 m on the Qinghai‐Tibet Plateau (Wang et al. 2023). Although Tibetan Plateau faces extreme environmental conditions with a soil with limited supply of nutrients (Li et al. 2024), wild barley has managed to thrive there for millennia, serving as a staple crop in early agricultural systems (Tang et al. 2021). Over thousands of years, both natural selection and human‐led breeding under challenging environments have led to the emergence of various adaptive traits in barley. The resulting genetic diversity provides a valuable foundation for ensuring future crop stability (Dawson et al. 2015). However, the loss of genetic variability due to selective breeding has made modern cultivated barley varieties more susceptible to numerous abiotic stresses (Schmidt et al. 2023), thus limiting the ability of breeders to develop cultivars resilient to diverse abiotic stress conditions.

Incorporating wild relatives into breeding efforts is a valuable strategy to broaden genetic diversity and improve tolerance to stresses, including P deficiency (Palmgren and Shabala 2024; Palmgren et al. 2015). Wild barley carries numerous ancestral or newly evolved alleles that help compensate for genetic losses from domestication bottlenecks over the past 10,000 years (Dawson et al. 2015). It holds significantly more genetic diversity than cultivated forms, with only about 40% of its alleles retained in modern barley cultivars (Kreszies et al. 2020; Nevo et al. 1979). Several studies have demonstrated that wild barley exhibits superior adaptation to low‐P stress, driven by distinct physiological, biochemical, and molecular mechanisms absent or diminished in cultivated varieties. These adaptations encompass differential protein profiles, enhanced root system architecture and rhizosphere dynamics, as well as specialized gene‐metabolite networks that collectively optimize P uptake and utilization (Nadira et al. 2014, 2016; Ye et al. 2018; Zhang et al. 2024). Despite advances in understanding molecular and physiological responses to P deficiency in wild barley, root morphological adaptations, particularly root hair traits, remain largely uncharacterized. Comparative analyses of root hair development under low‐P stress between wild and cultivated barley are notably limited, hindering efforts to link specific phenotypes to P acquisition efficiency.

To address this gap, the present study investigated the role of root hair traits in P acquisition under low‐P conditions across 24 barley genotypes, comprising 12 cultivated and 12 wild accessions with contrasting tolerance to low‐P conditions. Two experiments were conducted. Genotypes were classified as low‐P tolerant or sensitive based on their relative dry weight in a pot experiment grown under P‐deficient conditions. We then systematically examined their root hair number and length in relation to P concentration, content, and biomass accumulation to identify potential differences in root hair development between tolerant and sensitive groups and to determine the associations of these traits with adaptive responses to P limitation. By leveraging the natural diversity between wild and cultivated barley, we aimed to determine whether enhanced root hair development consistently supports enhanced P acquisition, or if its functional contribution depends on genotype‐specific responses to P deficiency. We reported that enhanced root hair number and length significantly increased shoot P concentration and content, as well as plant biomass, in low‐P sensitive barley genotypes, whereas low‐P tolerant genotypes maintained growth with little or even negative association with root hair traits. These findings showed that root hairs act as compensatory mechanisms for P acquisition in low‐P sensitive genotypes rather than universal indicators of P uptake efficiency, providing breeders with a clear basis for targeting wild germplasm to improve P efficiency while prioritizing alternative traits in tolerant lines. These insights will deepen our understanding of root morphological adaptations to nutrient stress and inform future breeding strategies for developing P‐efficient barley cultivars.

## 2 Materials and Methods

### 2.1 Plant Genotypes and Growth Conditions

Twenty‐four barley [12 cultivated ( *Hordeum vulgare* ) and 12 wild ( *Hordeum spontaneum* )] genotypes were grown under controlled greenhouse conditions at The University of Western Australia. Seeds were sown on June 8, and plants were harvested on August 1, 2024. The mean day/night temperatures were 22°C and 15°C, respectively; the relative humidity level was 65%; and the day length averaged ~10.5 h. Plants were cultivated in pots filled with growth medium consisting of agricultural topsoil collected from Cunderdin Agricultural College (0–15 cm depth) blended with sieved river sand in a 1:9 weight ratio. The basic physicochemical characteristics of the soil and sand were measured by the CSBP Future Analytical Laboratories (Bibra Lake, Australia), revealing phosphorus concentrations of 10 and 3 mg kg^−1^ in soil and sand, respectively (by the Colwell method). The detailed analyses of soil and sand properties are provided in Table S1. Each pot contained 1.1 kg of the soil mixture, and to prevent soil loss during watering, four layers of paper towel were placed at the bottom. Prior to sowing, the pots were thoroughly supplied with basal nutrient solution for 2 days to reach 80% of the soil's field capacity corresponding to approximately 12% moisture content by weight, using the already described method by Pang et al. (2017). The composition of the nutrient solution was as follows: 284.5 mg kg^−1^ Ca(NO_3_)_2_·4H_2_O, 244.7 mg kg^−1^ K_2_SO_4_, 42.98 mg kg^−1^ NH_4_Cl, 1.95 mg kg^−1^ CuSO_4_·5H_2_O, 8.86 mg kg^−1^ ZnSO_4_·7H_2_O, 0.68 mg kg^−1^ H_3_BO_3_, 1.95 mg kg^−1^ CuSO_4_·5H_2_O, 22.56 mg kg^−1^ FeNa‐EDTA, and 1.01 mg kg^−1^ NaMoO_4_·2H_2_O. Pots were divided into two groups: one receiving low‐P (5 mg kg^−1^) and the other receiving sufficient‐P (20 mg kg^−1^) treatment. Six healthy seeds were sown in each pot, and only two seedlings of uniform height were kept after 2 weeks. The pots were supplied with an equal volume of nutrient solution every 3 days to maintain soil moisture at the optimal level (12% w/w).

### 2.2 Sampling and Data Collection

After 54 days of sowing, plants were harvested at the vegetative stage to assess biomass accumulation and P concentration. Shoots and roots were separated, gently washed with deionized water to remove residual soil, and oven‐dried at 60°C for 48 h. After drying, shoot and root dry weights were recorded to assess total biomass accumulation under different P regimes.

### 2.3 Phosphorus Concentration Analysis

Dried shoot samples were ground into a fine powder, and 0.15 g of each sample was transferred into a 25 mL Erlenmeyer flask. Acid digestion was carried out by adding 3 mL of concentrated nitric acid to each flask, followed by heating at 100°C for 20 min, while carefully avoiding overflow. Once the brown fumes had dissipated and no visible particulate matter remained in the solution, the flasks were allowed to cool for 5 min. Subsequently, 0.5 mL of concentrated perchloric acid was added, and the flasks were heated at 125°C until the digest turned colourless. To dehydrate any residual silica, the digests were further heated at 170°C. After digestion, the flasks were cooled to room temperature for 10 min, and 4 mL of Milli‐Q water was added to each flask to dilute the samples.

Phosphorus concentration in digested shoot samples was determined using the molybdenum blue colorimetric method. A freshly prepared colour reagent was used for the molybdenum blue reaction, prepared by adding 0.16 g of L‐ascorbic acid to a composite solution containing ammonium molybdate, sulfuric acid, and antimony potassium tartrate, as previously described (John 1970) with slight modifications. A 10 μL of the digested sample was pipetted into a 96‐well microplate, followed by 48 μL of ascorbic acid solution, and the reaction volume was topped up to 300 μL using Milli‐Q water. The solution was mixed thoroughly and left to react at room temperature for 10 min to allow the development of blue colour. Absorbance was measured at 882 nm using a PerkinElmer EnSight multimode plate reader, and P concentration was quantified using a standard curve prepared from known KH_2_PO_4_ concentrations. Final values were expressed as milligram P per gram of dry shoot tissue. The shoot P content and phosphorus acquisition efficiency (PAE) were calculated by using the following formulas, respectively:

ShootPcontent=shootPconcentration×shootdryweight

PAE=root hair length/shootPcontent

### 2.4 Root Hair Quantification Under Contrasting Phosphorus Conditions

To evaluate root hair traits under low‐P treatment, seeds of all genotypes were surface sterilized using 10% bleach for 10 min followed by rinsing them 5–6 times with distilled water. Then the seeds were placed on sterile Whatman filter paper moistened with either low‐P (0.3 μM KH_2_PO_4_) or sufficient‐P (30 μM KH_2_PO_4_) solution prepared in deionized water. The filter paper was placed in Petri dishes and incubated at 25°C in the dark for 3 days to promote uniform germination.

An individual root was selected from each seedling for microscopic examination. Photographs of root hairs were captured with the help of Leica DM500 fluorescence microscope (Leica Microsystems), which was fitted with DFC295 digital colour camera. The number of root hairs along the entire root and the root hair length were measured using the ImageJ software (Rasband 2010).

For analysis of root hairs in sand‐soil substrate, four contrasting genotypes (two cultivated—Macquarie and YSM3; two wild—X165 and X61) were selected based on their extreme root hair phenotypes. Plants were grown for almost 8 weeks under low‐P (5 mg kg^−1^) or adequate‐P (20 mg kg^−1^) supply. At harvest, roots were gently washed with deionized water to remove adhering substrate. Root hairs were imaged from the maturation zone, and root hair density was determined by counting all hairs along a 1‐mm segment, while root hair length was measured from 15 to 20 randomly selected hairs in the same region. Measurements were obtained from 3 to 4 biological replicates per genotype per P treatment.

### 2.5 Statistical Analysis

All data underwent statistical analysis using the software IBM SPSS Statistics (version 29.0) and statistical significance was calculated at thresholds of *p* ≤ 0.05 and *p* ≤ 0.01. All figures display mean values, with error bars representing the standard error of the mean (SEM). Graphs and plots, including correlation analysis, were generated using Microsoft Excel (Microsoft 365, version 2502) and GraphPad Prism (version 10.0).

## 3 Results

**FIGURE 1:**
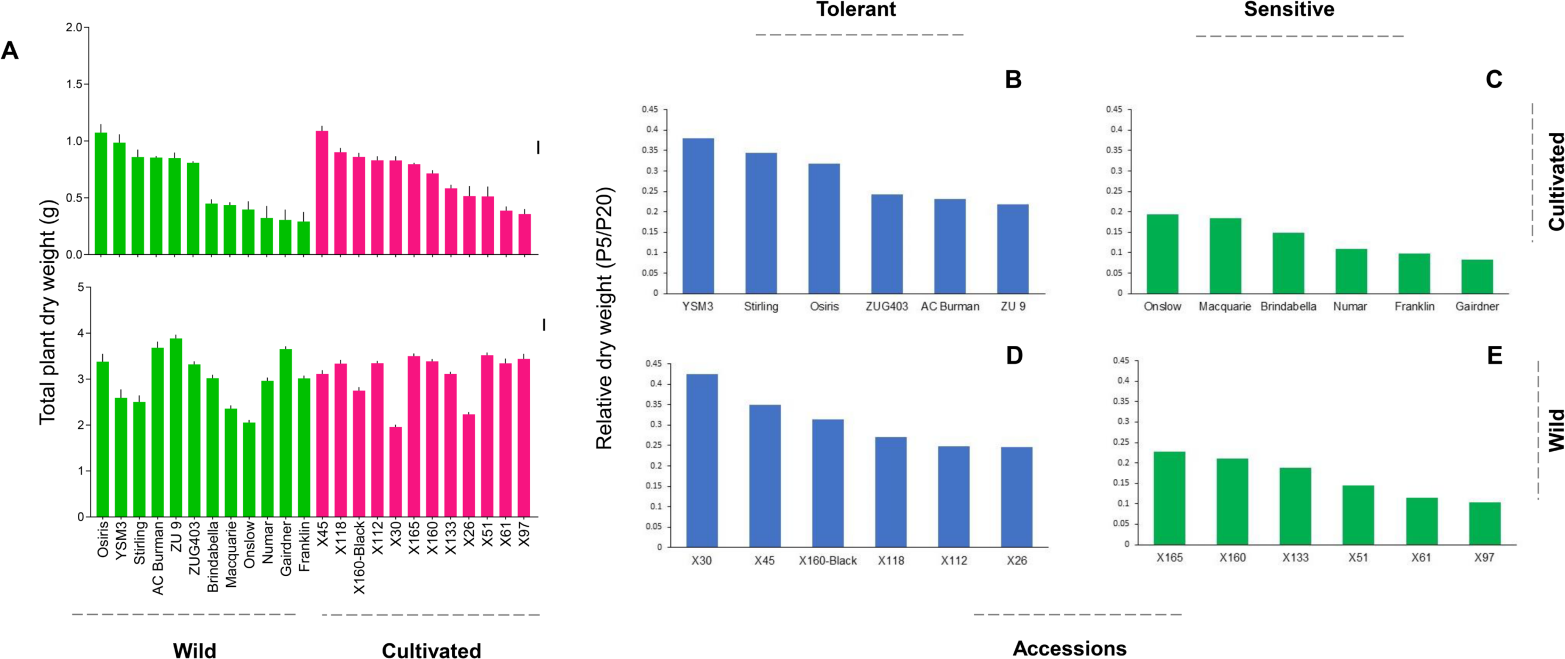
Differential growth response of cultivated and wild barley accessions to low and high phosphorus supply. (A) Absolute total dry weight of cultivated and wild accessions grown under low‐phosphorus (low‐P; 5 mg kg^−1^ P) and high‐phosphorus (high‐P; 20 mg kg^−1^ P) conditions. Data are presented as mean ± SE (*n* = 5–6). Black vertical bars indicate the LSD_0.05_ for genotype comparisons. (B–E) Relative dry weight (P5/P20), calculated as the ratio of total dry weight under low‐P (P5) to that under high‐P (P20), for (B) cultivated tolerant, (C) cultivated sensitive, (D) wild tolerant, and (E) wild sensitive accessions.

A significant genetic variability in response to contrasting phosphorus regimes was observed in both cultivated and wild barley accessions (Figure 1). When grown under low P (P5, 5 mg kg^−1^ P) compared with high P (P20, 20 mg kg^−1^ P), relative total dry weight (P5/P20) varied markedly among accessions, ranging from ~0.1 in the most sensitive lines to > 0.4 in the tolerant ones (Figure 1B–E). This large variation indicates that some genotypes were able to sustain a substantial proportion of their biomass even under severe P limitation, whereas others showed pronounced growth reduction. Based on these responses, we have set up a relative threshold of 0.25 as a proxy for stress tolerance. Genotypes that showed a reduction in biomass of over 75% (relative dry weight < 0.25) were classified as sensitive; those with relative DW > 0.25 as tolerant. Sensitive genotypes, especially wild barley, showed strong correlations between root hair traits and multiple performance indicators, including shoot P concentration, shoot P content, and biomass, reflecting a greater reliance on root hair development. In tolerant lines, these associations were weak or absent, suggesting that other mechanisms support P acquisition and growth under deficiency. Collectively, the results highlight that root hair contribution to P uptake efficiency is genotype‐dependent and interacts with both growth and nutritional outcomes.

### 3.1 Root Hair Growth Is Regulated by Phosphorus Availability in Barley

**FIGURE 2:**
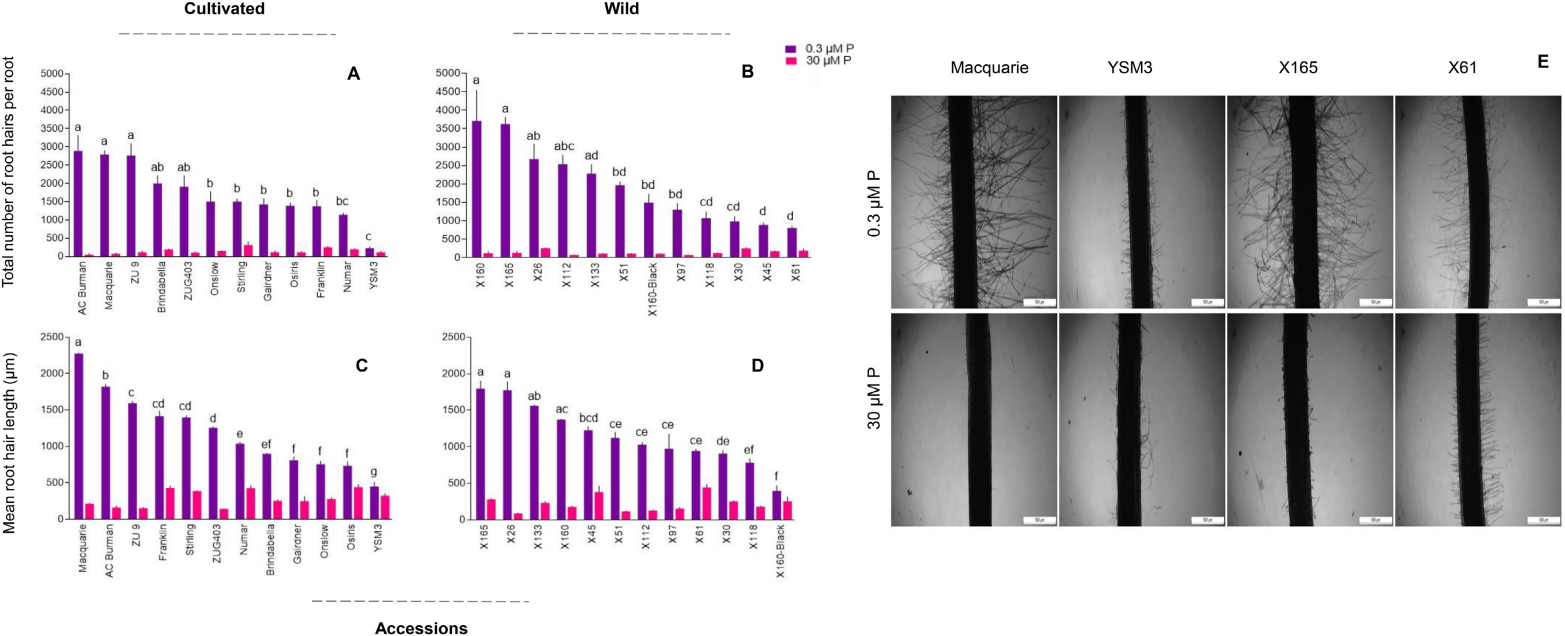
Root hair responses of cultivated and wild barley genotypes to low and high phosphorus (P) availability. (A and B) Total number of root hairs along the whole individual root, (C and D) mean root hair length (μm) in (A and C) cultivated and (B and D) wild barley genotypes grown under low‐P (0.3 μM) and high‐P (30 μM) conditions. Genotypes are arranged in descending order based on their mean values under low‐P. Bars represent mean ± standard error of the mean (SEM) with *n* = 3–4 biological replicates. Different letters above bars indicate significant differences (one‐way ANOVA with Tukey's post hoc test, *p* < 0.05). (E) Representative root hair images of selected cultivated (Macquarie and YSM3) and wild (X165 and X61) genotypes under low‐P (top row) and high‐P (bottom row) conditions. Images were taken from the maturation zone, approximately 3 mm behind the root tips. Scale bars = 500 μm.

Exposure to contrasting P regimes revealed marked genotypic variation in root hair development across both cultivated and wild barley accessions. While low‐P conditions generally stimulated root hair proliferation, the extent and nature of this response varied substantially across genotypes. All genotypes were systematically ranked in descending order based on their root hair number and length under P‐deficient conditions (Figure 2A–D). Representative images (Figure 2E) highlight pronounced root hair development under low and sufficient P availability in selected genotypes of both cultivated and wild barley.

Interestingly, genotypes characterized by inherently sparse or shorter root hairs under sufficient‐P conditions exhibited the most pronounced increases in the root hair number and length when subjected to P‐deficiency. Root hair responses to P‐deficiency varied not only in magnitude but also in the dominant trait involved; whether through increased number, elongation, or a combination of both. Under sufficient P supply (30 μM), most accessions maintained low to moderate root hair number and short root hairs, with limited variability among genotypes. However, exposure to low P triggered divergent morphological responses across accessions. In a subset of cultivated genotypes, root hair number and length increased simultaneously, resulting in dense and elongated root hairs under deficiency. This pattern was especially evident in barley cultivars AC Burman, Macquarie, and ZU 9 that already showed moderate root hair number and length under high P but responded with a significant increase in both traits, positioning them among the highest for both number and length under low‐P (Figure 2A,C). Root hair responses among wild barley accessions under P deficiency revealed a high degree of morphological variability, reflecting distinct trait expression patterns across genotypes. While low P consistently induced some level of root hair development, the extent and nature of this induction varied markedly among the genotypes. A subset of wild barley accessions, including X165 and X26, exhibited strong plasticity in both root hair number and length, with low values under sufficient P transitioning to among the highest observed under deficiency (Figure 2B,D). These accessions represent dual‐trait responders, capable of simultaneously enhancing surface area through both initiation and elongation mechanisms.

In contrast, wild barley, such as X133 and X112, displayed a steeper increase in root hair number than in length (Figure 2B,D), indicating a response skewed toward higher initiation density rather than elongation. This pattern suggests differential trait prioritization, where expansion of total absorbing surface may be achieved predominantly through proliferation rather than individual hair extension. Meanwhile, accessions like X61 showed consistently low root hair number and length across both P regimes, indicating a limited capacity or insensitivity to modulate root epidermal traits in response to external P availability.

### 3.2 Root Hair Number Negatively Correlates With Tolerance to Phosphorus Deficiency in Barley

**FIGURE 3:**
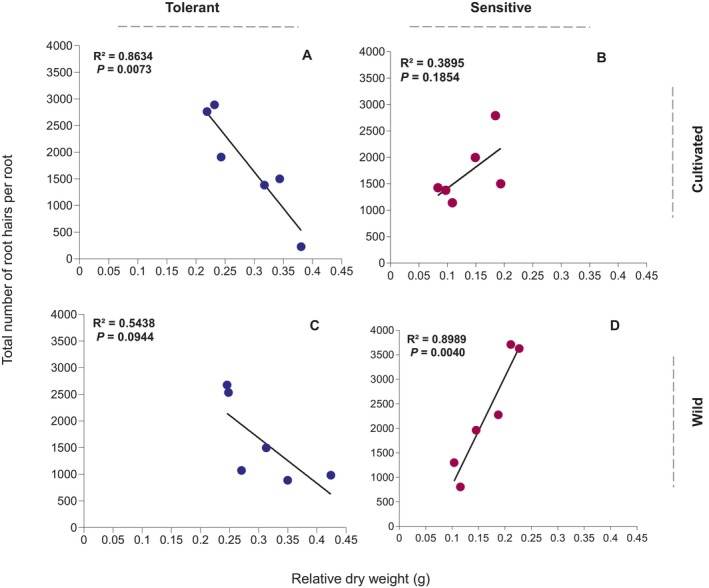
Correlation between root hair number and relative dry weight in cultivated and wild barley genotypes under low‐phosphorus condition. Scatter plots showing genotype‐wise associations between total root hair number (counted along the entire root of each replicate at fourth day after growing in Petri dish) and relative total dry weight in barley genotypes classified as (A and C) low phosphorus‐tolerant and (B and D) low phosphorus‐sensitive. Each bullet point represents an individual barley genotype. Linear regression lines with corresponding *R* ^2^ and *p*‐values indicate the strength of the correlation within each group.

We correlated patterns of root hair development with plant tolerance to low‐P stress, dividing all accessions into low‐P tolerant (T) and low‐P sensitive (S) groups on the basis of their relative dry weight (Figure 1B–E). Under low P supply (0.3 μM KH_2_PO_4_), a strong negative correlation (*R* ^2^ = 0.863, *p* < 0.05) was observed between whole‐root root hair count and relative dry weight in the cultivated T group (Figure 3A), suggesting that T genotypes maintained growth with fewer root hairs, potentially relying on alternative P acquisition or utilization strategies. In contrast, no significant correlation was found in the cultivated S group (Figure 3B), indicating that variation in root hair traits may not directly influence plant biomass under low P in this group. The same trend was also observed for wild barley accessions: a negative correlation in the tolerant cluster (Figure 3C), and a highly significant positive correlation (*R* ^2^ = 0.898, *p* < 0.005; Figure 3D) in the sensitive group. Taken together, these data suggest that the development of root hairs under low‐P conditions is more likely a compensatory response to P deficiency in S genotypes, a strategy to cope with low‐P conditions.

### 3.3 Root Hair Traits Show Contrasting Associations With Shoot Phosphorus Status Across Barley Genotypes

**FIGURE 4:**
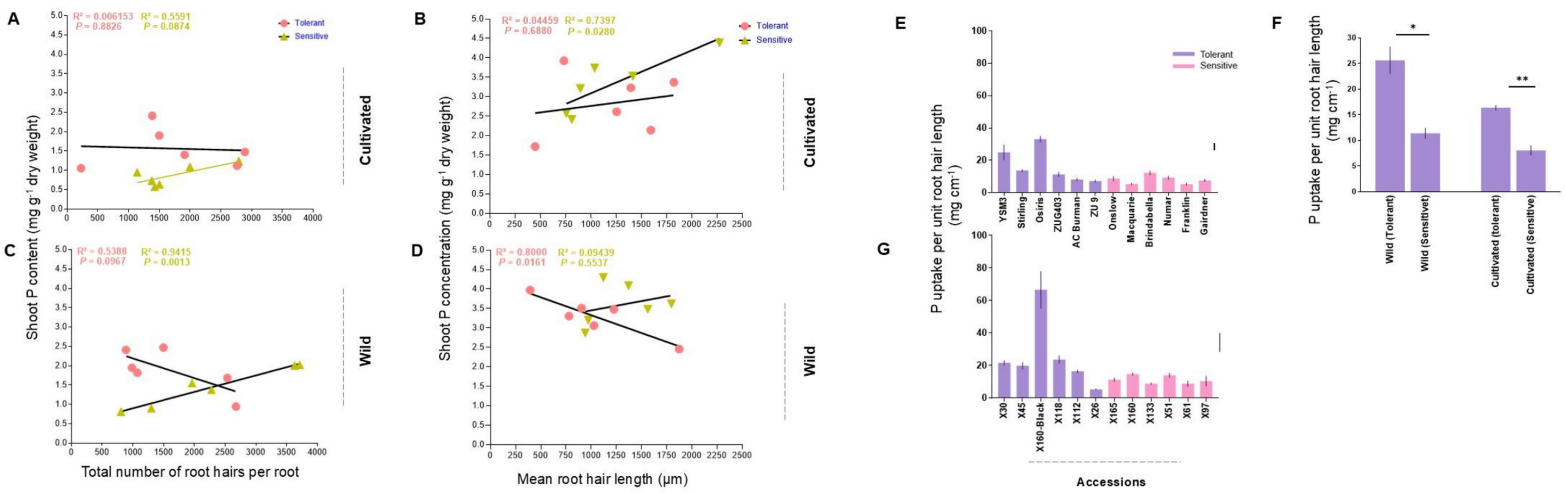
Relationships between root hair traits, shoot phosphorus status, and phosphorus acquisition efficiency (PAE) in cultivated and wild barley genotypes under low‐phosphorus condition. (A–D) Correlations between shoot P concentration or content and root hair length or number in (A) cultivated tolerant, (B) cultivated sensitive, (C) wild tolerant, (D) and wild sensitive genotypes. Each point represents an individual genotype. Linear regression lines are presented with corresponding *R* ^2^ and *p*‐values. (E–G) Phosphorus uptake per unit root hair length (mg g^−1^) for tolerant and sensitive accessions with vertical LSD_0.05_ bars indicating statistical separation, (F) mean PAE per unit root hair length for tolerant and sensitive groups within wild and cultivated barley (*p* < 0.05).

Root hair traits showed contrasting associations with shoot P status across barley genotypes (Figure 4). Compared to cultivated T genotypes that had no significant associations with shoot phosphorus, S genotypes displayed moderate to strong positive relationships between shoot P metrics and both total root hair number and mean root hair length (Figure 4A,B), indicating that greater root hair development corresponded to improved P nutrition. In wild barley, these associations diverged even more markedly. Wild tolerant (WT) genotypes exhibited weak to negative relationships between shoot P status and root hair traits (Figure 4C,D), whereas wild sensitive (WS) genotypes showed a strong positive association between root hair number and shoot P concentration (*R* ^2^ = 0.942; *p* < 0.01). These contrasting patterns demonstrate that the functional relevance of root hair traits is highly genotype‐specific.

A clear genotypic variation was observed in phosphorus acquisition efficiency (PAE) per unit root hair length. Wild barley showed the greatest functional diversity with tolerant genotypes such as X160‐Black and X118 showing the highest efficiencies, substantially exceeding the values of cultivated genotypes (Figure 4G). Compared with wild barley, cultivated genotypes showed a compressed distribution in which tolerant and sensitive lines overlapped (Figure 4E). This narrower range reflects reduced functional diversity in root hair efficiency within cultivated germplasm. These findings suggest that wild barley possesses superior root hair‐mediated phosphorus uptake capacity and that enhanced P acquisition per unit root hair length is a major contributor to tolerance under low‐P conditions in this group.

Group‐level mean PAE showed clear differences across domestication and tolerance classes (Figure 4F). Wild tolerant genotypes exhibited the highest mean efficiency, nearly doubling the values of WS lines, indicating a substantially greater efficiency of individual root hairs in acquiring P under low‐P condition. Cultivated barley showed the same directional pattern, with tolerant lines outperforming cultivated sensitive lines, but the separation between classes was smaller. This narrower range in cultivated barley suggests a more constrained functional capacity of root hairs compared with the broader diversity observed in wild germplasm. Together, these patterns indicate that tolerant genotypes use more physiologically efficient strategies for P uptake, whereas sensitive lines primarily rely on compensatory morphological responses.

### 3.4 Root Hair Traits Enhance Biomass Accumulation in Low‐P Sensitive but Not in Tolerant Wild Barley Genotypes

**FIGURE 5:**
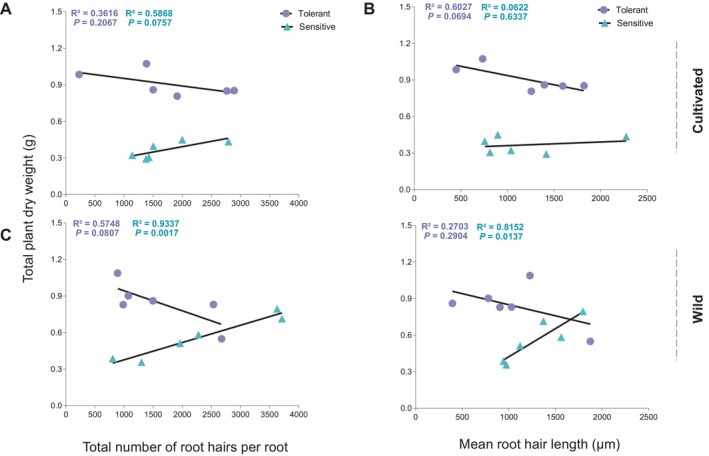
Correlation between plant dry weight and root hair traits in cultivated and wild barley genotypes under low‐phosphorus conditions. Total plant dry weight for cultivated and wild barley genotypes was plotted against (A and C) total number of root hairs per individual root and (B and D) mean root hair length. Linear regression lines are shown with corresponding *R* ^2^ values indicating the proportion of variance explained, and *p*‐values denoting statistical significance.

Among cultivated genotypes, both total root hair number and mean root hair length exhibited weak to moderate negative associations with shoot dry weight in T lines (Figure 5A,B). These negative slopes indicate that, for tolerant cultivated genotypes, increasing the number or length of root hairs does not contribute to greater biomass and may instead reflect a compensatory developmental response when other P‐uptake mechanisms are already effective. Sensitive cultivated genotypes showed minimal or non‐significant correlations for both traits (Figure 5A,B), suggesting that in cultivated backgrounds, root hair traits are generally poor predictors of biomass accumulation under P deficiency.

In wild barley, however, the pattern was far more pronounced and biologically meaningful. WS genotypes displayed strong and statistically significant positive correlations between biomass and both root hair length and number (*R* ^2^ = 0.82–0.9; Figure 5C,D). These relationships indicate that increases in root hair production and elongation directly support biomass accumulation in these genotypes, highlighting a strong reliance on root hair development to improve growth under low‐P stress. Conversely, WT genotypes showed weak or negative slopes for both traits (Figure 5C,D), consistent with reduced dependence on root hair morphology and greater reliance on alternative or more physiologically efficient P‐acquisition strategies.

### 3.5 Root Hair Traits Show Contrasting Relationships With Root Biomass in Cultivated and Wild Barley

**FIGURE 6:**
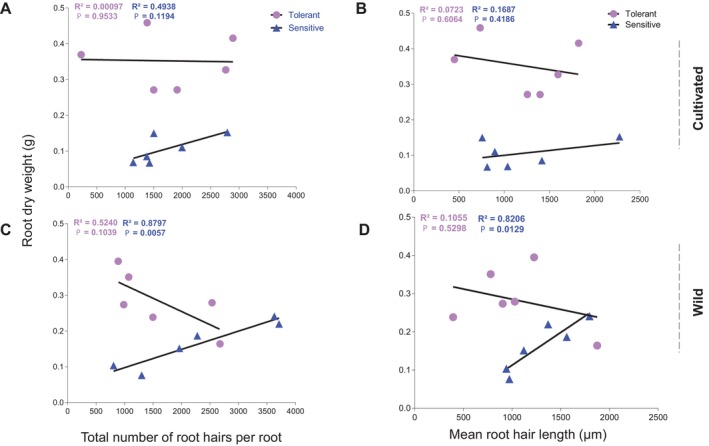
Correlation between root dry weight and root hair traits in cultivated and wild barley genotypes under low‐phosphorus conditions. (A–D) Plots illustrate how variation in root dry weight aligns with differences in (A and C) root hair number and (B and D) mean root hair length in cultivated and wild barley genotypes classified as tolerant or sensitive to low‐P. Each circle represents an individual genotype. Regression lines are fitted to visualize trends. Coefficient of determination (*R* ^2^) and *p*‐values are provided to indicate the strength and significance of the relationships, respectively.

Variation in root hair traits among barley genotypes revealed distinct and contrasting associations with root biomass under P‐deficient conditions, reflecting divergent adaptive responses between cultivated and wild genotypes (Figure 6). In cultivated barley, weak and inconsistent associations between root biomass and root hair traits (Figure 6A,B) indicate that enhanced root hair development does not substantially contribute to root system growth in these lines.

In a marked contrast, the relationships were more pronounced and biologically meaningful in wild barley. The WT group showed moderate negative correlations between root hair number or length and root biomass (Figure 6C,D), suggesting that increases in root hair development may occur as a compensatory response to reduced root growth rather than as a driver of biomass accumulation. WS genotypes exhibited strong and statistically significant positive correlations for both root hair number and length (*R* ^2^ = 0.87 and 0.83–0.88 significant at *p* < 0.05; Figure 6C,D). These results indicate that, in wild sensitive lines, greater investment in root hair development is closely linked to enhanced root biomass under low‐P conditions.

### 3.6 Cumulative Root Hair Length Strongly Predicts Biomass in Wild Sensitive Barley

**FIGURE 7:**
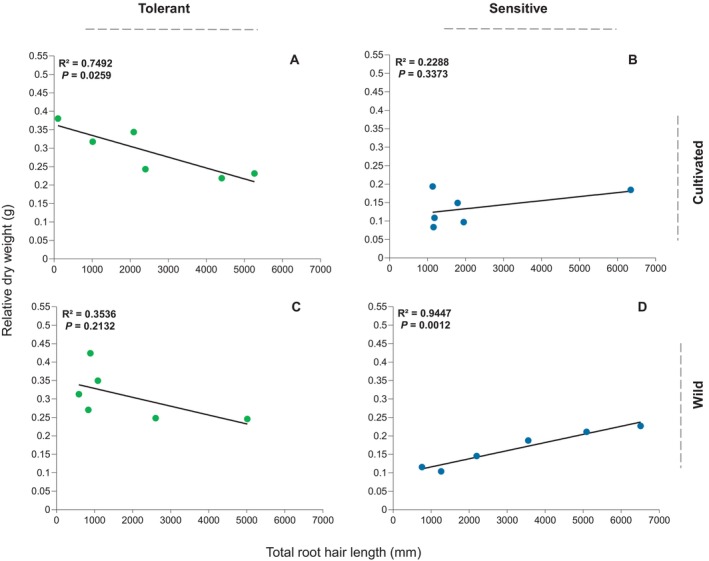
Relationship between total root hair length and relative dry weight in barley genotypes under low‐phosphorus conditions. The plots show linear relationships between cumulative root hair length and relative dry weight in barley genotypes grouped by phosphorus sensitivity (A and C: Tolerant; B and D: Sensitive) and domestication status (A and B: Cultivated; C and D: Wild). Each circle represents a single genotype. Regression lines illustrate the relationship, with corresponding R^2^ and *p*‐values indicating the strength and significance of the correlation.

To capture the combined effect of root hair proliferation and elongation, we calculated cumulative root hair length per root (mean root hair length × number of root hairs). This integrative trait provided a clearer view of how epidermal investment relates to plant growth under P deficiency. A significant negative association between total root hair length and biomass was observed in cultivated T lines (Figure 7A), while wild T genotypes showed only a weak, non‐significant trend in the same direction (Figure 7C), suggesting that additional root hair investment did not confer growth advantages in these backgrounds but rather represented a costly response. Compared to cultivated S lines, which showed a weak and statistically non‐significant relationship between both traits (Figure 7B), wild S genotypes showed a strikingly strong and significant positive correlation (*R* ^2^ = 0.94, *p* < 0.01), highlighting cumulative root hair length as a decisive factor supporting biomass maintenance under low‐P conditions. This finding underscores the functional relevance of epidermal traits, particularly in stress‐sensitive backgrounds, whereas tolerant lines appear to rely on alternative strategies.

### 3.7 Validation of Root Hair Traits in Sand‐Soil System

**FIGURE 8:**
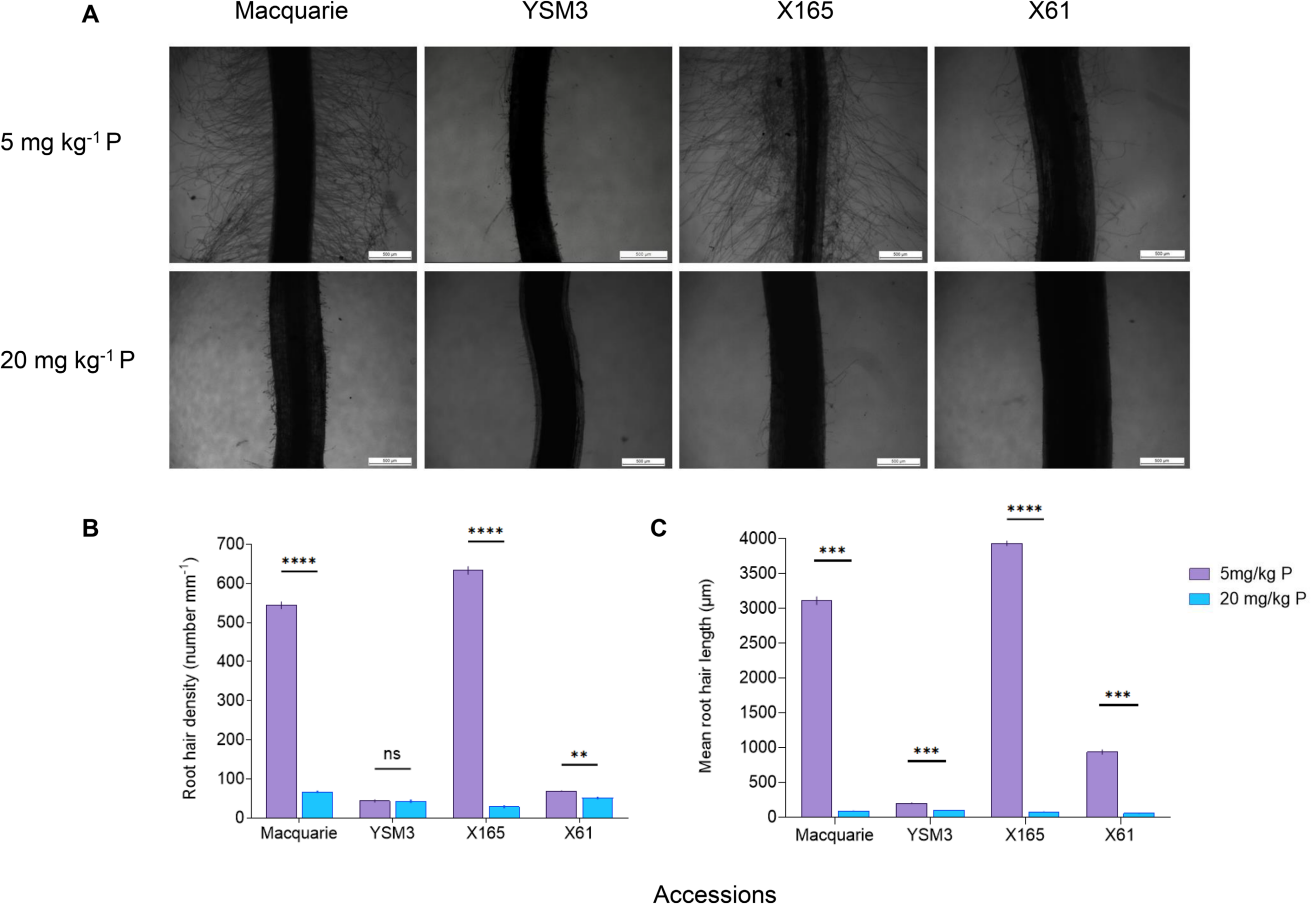
Root hair responses of selected 8‐week‐old barley genotypes grown in a sand‐soil substrate under contrasting phosphorus levels. (A) Representative micrographs of root hairs from two cultivated (Macquarie and YSM3) and two wild (X165 and X61) barley genotypes grown under low‐P (5 mg kg^−1^) and adequate‐P (20 mg kg^−1^) conditions. Images show the maturation zone approximately 3 mm behind the root tip. Scale bars = 500 μm. (B) Root hair density (number mm^−1^) of the four genotypes under low‐ and adequate‐P supply. (C) Mean root hair length (μm) under both P levels. Bars represent mean ± SE. Statistical differences between P treatments within each genotype were determined using Student's *t*‐test (ns, not significant; *p* < 0.01; *p* < 0.001; *p* < 0.0001).

Microscopy images of seedlings grown in sand‐soil mixture revealed distinct P‐dependent response in the root hair development (Figure 8A). Under low‐P (5 mg kg^−1^) supply, all tested genotypes produced visibly more and longer root hairs, whereas high‐P (20 mg kg^−1^) suppressed root hair formation and growth. Quantitative measurements confirmed that Macquarie produced approximately 12‐fold denser root hairs and 15‐fold longer root hairs than YSM3, under low‐P supply (Figure 8B,C). Similarly, X165 (selected as a high root hair producer from the filter paper screen) exhibited a profound increase in both root hair number and length, with approximately 9 times more and 4 times longer root hairs compared to X61 (Figure 8B,C). Overall, these results demonstrate that genotype‐specific root hair growth patterns initially observed in the filter‐paper assay are stable and reproducible when seedlings are grown in a sand and soil mixture. This further supports the filter‐paper assay as a reliable predictor of genotype performance under more realistic growth conditions.

## 4 Discussion

In this study, we have analysed root hair responses to P availability in a genetically diverse panel of barley (cultivated and wild) genotypes with contrasting P tolerance. While root hair traits exhibited substantial genotypic variation, their association with biomass and P accumulation was strongly genotype‐dependent and far from universally beneficial. Moreover, in both cultivated and wild low‐P‐tolerant (T) genotypes, the association between root hair development and plant performance (in terms of plant growth and P accumulation) was negative, challenging the notion that longer, denser root hairs will automatically benefit plant adaptation to low‐P soils. Instead, our data reveal that the functional relevance of root hair traits in P acquisition varies substantially among barley genotypes and is strongly shaped by their inherent tolerance to P deficiency. These findings are consistent with recent evidence showing that enhanced root hair length does not necessarily improve P uptake efficiency (Zhang et al. 2018).

On the other hand, in the low‐P sensitive (S) genotypes, greater epidermal investment was closely associated with improved shoot biomass, root biomass, and shoot P accumulation. This is consistent with previous work demonstrating the importance of root hairs as adaptive structures for resource capture in low‐input environments (Gahoonia and Nielsen 1998; Gahoonia et al. 2001; Haling et al. 2013; Itoh and Barber 1983). This supports the view that epidermal plasticity can serve as a compensatory strategy in genotypes with limited P‐uptake efficiency, consistent with recent findings emphasizing root hairs as a key component of stress adaptation in low‐input environments (Gahoonia and Nielsen 1998; Gahoonia et al. 2001; Haling et al. 2013; Itoh and Barber 1983; Zhao et al. 2025).

The apparent disconnect between root hair traits and biomass accumulation in T (both wild and cultivated) genotypes (Figure 5A–D) raises important questions about the functional relevance of root hairs in these backgrounds. In such cases, increased root hair formation may reflect a trade‐off in which metabolic resources are allocated to epidermal traits without a corresponding functional gain in nutrient acquisition. This interpretation aligns with previous findings (Nestler and Wissuwa 2016), which revealed that enhanced root hair traits in rice did not consistently confer improved P efficiency, reflecting the need to interpret such traits within a broader functional framework that accounts for genotype‐specific adaptive strategies.

Root hair proliferation enhances the root‐soil interface by enabling roots to exploit otherwise inaccessible P pools (Haling et al. 2013). Soil‐based experiments using ^32^P‐labeled nutrient hotspots (accessible exclusively to root hairs) have demonstrated that root hairs alone can supply over 60% of a plant's total P requirement in rye plants (Gahoonia and Nielsen 1998). However, the present study provides compelling evidence that the relationship between root hair traits and P uptake is not evolutionarily or functionally conserved across barley genotypes. Weak or negative correlations between root hair number or length and shoot P status in the T lines (Figure 4A–D) depict that these genotypes maintain P uptake through mechanisms (such as enhanced phosphatase activity, stronger P mobilization in the rhizosphere, or high‐affinity Pi transporters) that operate largely independently of root hair morphology.

In S genotypes (especially the wild barley), the strong positive associations between root hair traits and shoot P status (Figure 4C,D) indicate a clear reliance on epidermal investment to acquire phosphorus under stress. This unexpected trend calls into question the commonly held notion that enhanced root hair growth is a consistently beneficial adaptation for improving P uptake. One plausible explanation is that excessive investment in root hair initiation may impose a metabolic or structural cost without proportionate returns in nutrient uptake, particularly when spatial placement or synchrony with rhizospheric P availability is suboptimal. Alternatively, genotypes with inherently poor capacity for P uptake may have upregulated genes controlling root hair formation as a compensatory, yet ineffective mechanism, in response to stress, ultimately resulting in inverse correlation.

From the breeding perspective, it is therefore essential to consider not merely the development of root hair traits themselves, but whether these traits translate into higher phosphorus acquisition efficiency (PAE) or not. PAE can serve as an important lens to understand the contrasting strategies of cultivated and wild barley. The exceptionally high PAE values observed in wild T genotypes (Figure 4G) point to a fundamentally more effective coupling between epidermal investment and nutrient capture in this germplasm. Rather than merely producing longer or denser root hairs, these genotypes appear capable of extracting substantially more phosphorus per unit root hair length, reflecting a deeper integration of epidermal traits with rhizosphere processes and physiological uptake pathways. In cultivated barley, by contrast, the narrow, overlapping range of PAE values between T and S groups suggests that domestication may have constrained the functional diversity of epidermal traits, limiting the extent to which root hair development can contribute to nutrient acquisition. Although sensitive lines depend more directly on root hair development to sustain internal P levels, this strategy comes at a cost: despite producing more or longer hairs, each unit of root hair is less efficient, as reflected in their lower PAE values compared with tolerant backgrounds. While it may be tempting to assume that increased root hair number and length reflect an adaptive strategy, our findings suggest that this trait alone is not a reliable proxy to cope with P uptake efficiency.

Biomass accumulation is a reliable indicator of P‐use efficiency (Veneklaas et al. 2012), yet the underlying morphological contributors, such as root hair density and length, exert genotype‐specific effects. In the wild S genotypes, our data revealed that traits like root hair number and elongation showed a positive correlation (Figure 5C,D) with plant dry biomass, suggesting that enhanced epidermal growth may serve as a compensatory strategy to maintain nutrient acquisition when other acquisition strategies are scarce. Root hairs, due to their relatively low metabolic cost compared to major architectural changes (Jakobsen et al. 2005), may offer a rapid and resource‐efficient means to enhance root‐soil contact and exploit localized P pockets. In the S genotypes, elongation and proliferation of root hairs could partially compensate for limitations in other below‐ground traits, thereby facilitating P acquisition and sustaining biomass production. This interpretation aligns with the “rhizoeconomics” framework (Lynch et al. 2005), which posits that investment in metabolically cheaper traits like root hairs becomes advantageous primarily when more carbon‐intensive strategies are either limited or ineffective. On the other hand, the T genotypes (Figure 5A–D), which showed no clear correlation between root hair traits and biomass, may rely more on integrated strategies such as deeper rooting, greater root branching, carboxylate exudation, or enhanced symbiosis, rendering the marginal benefits of additional root hair investment negligible. This divergence highlights how the functional significance of root hairs is not uniform across genotypes and may depend on the underlying root system architecture and metabolic trade‐offs.

Root dry weight serves as a critical proxy for belowground investment under P deficiency and reveals genotype‐specific strategies of adaptation. In the present study, a significant positive correlation between root hair traits and both root biomass (Figure 6C,D) as well as total shoot P content (Figure C and D) exclusively in the wild S genotypes suggests that enhanced root hair development plays an active role in supporting P uptake, accompanied by larger root growth in nutrient‐limited environments. Auxin‐mediated signaling may underlie this coordination between root hair length and root biomass. It has been demonstrated that low‐P conditions stimulated indole‐3‐acetic acid levels via PIN‐mediated auxin transport, leading to increased expression of root‐hair‐specific transcription factors (e.g., root hair defective six‐like 2/4, RSL2/RSL4) and promoting downstream root development (Bhosale et al. 2018), suggesting that root hairs can trigger systemic root growth responses under P deficiency. However, confirming such regulatory mechanisms will require targeted physiological and molecular analyses in future studies, particularly to dissect the role of hormone signaling in genotype‐specific foraging strategies under low‐P stress.

Our analysis of cumulative root hair length, combining both proliferation and elongation, further emphasized the genotype‐dependent role of root hairs under P‐starved conditions. In cultivated T lines (Figure 7A), the negative association between cumulative root hair length and relative dry weight reflects not merely a lack of benefit but a potential cost, where additional epidermal proliferation may have diverted resources from alternative strategies that are inherently more effective in these genotypes. A near‐perfect positive correlation between cumulative root hair length and relative dry weight (Figure 7D) highlights the decisive role of epidermal proliferation in sustaining growth under P deficiency. As cumulative root hair length effectively represents the total absorptive surface area available for soil contact, this association suggests that wild sensitive lines rely heavily on expanding the root–soil interface to mobilize immobile P. Such a strategy is consistent with empirical evidence showing that the presence of root hairs improves root–soil contact and enhances P acquisition in barley across contrasting soil environments. This interpretation is supported by physiological evidence from barley, where wild‐type barley plants extracted nearly twice the amount of P from the rhizosphere compared with the root‐hairless mutant, with a P depletion zone extending approximately equal to the length of root hairs. Under low‐P conditions, only the wild type maintained growth, underscoring the essential role of root hairs in P acquisition (Gahoonia and Nielsen 2003).

Together, our data illustrate that although low P consistently promotes root hair development in barley, the dominant contributing trait, whether root hair number, length or both, varies across genotypes and is not strictly determined by subspecies. These patterns underscore the complex and genotype‐specific nature of root surface remodelling under P stress. The robustness of these conclusions is further supported by the observation that genotypic contrasts in root hair traits were maintained even under the sand‐soil conditions (Figure 8) used for genotype screening, underscoring that these differences reflect intrinsic phenotypic patterns rather than assay‐specific artefacts. A more nuanced understanding of how different root phenes interact across genetic backgrounds is essential for effective trait‐based breeding targeting enhanced P‐use efficiency.

## Author Contributions

S.S. conceived the research idea and supervised the overall project. T.N. performed sand‐soil experiment for root hair analyses, microscopic and biochemical analyses, interpreted data, and drafted the manuscript with input from S.S. and J.P. Y.T. conducted glasshouse experiments and analysed data. J.P. provided additional supervisory guidance and assisted with soil and sand physiochemical analyses. M.Z., F.Z., and Z.‐H.C. offered further supervisory input, critical feedback, and manuscript editing. All authors contributed to revisions and approved the final version.

## Funding

This work was supported by Australian Research Council, LP210200955.

## Supporting information


**Table S1:** Physicochemical properties of soil and river sand used for growth of barley genotypes.

## Data Availability

Data will be made available on request.
