# Supplementary material for: Reassessing the Role of Root Hairs in Adaptation to Low Phosphorus Conditions: Lessons From Wild Barley
Source: Physiol Plant. 2026 Aug 11;178(4):e71062. doi: 10.1111/ppl.71062 (PMC13458639; doi:10.1111/ppl.71062)
Supplement: Supplementary file 1 [file PPL-178-e71062-s001.pdf]

Supplementary Data of the Article

Reassessing the Role of Root Hairs in Plants Adaptation to Low Phosphorus Conditions: Lessons from Wild Barley

Tahmina Nazish[1], Yunpeng Tao[1], Jiayin Pang[1,2], Meixue Zhou[3], Fanrong Zeng[4], Zhong-Hua Chen[5], Sergey Shabala[1,6,*]

[1]School of Biological Sciences, The University of Western Australia, Perth, WA, 6001, Australia

[2]The UWA Institute of Agriculture, The University of Western Australia, Perth, WA, 6001, Australia

[3]Tasmania Institute of Agriculture, University of Tasmania, Hobart, TAS 7001, Australia

[4]MARA Key Laboratory of Sustainable Crop Production in the Middle Reaches of the Yangtze River, College of Agriculture, Yangtze University, Jingzhou, China

[5]School of Agriculture, Food and Wine, Waite Research Institute, University of Adelaide, Glen Osmond, SA 5064, Australia

[6]International Research Centre for Environmental Membrane Biology, Foshan University, Foshan 528000, Guangdong, China

[*]Corresponding author; Email: sergey.shabala@uwa.edu.au

Supplementary Table 1. Physicochemical properties of soil and river sand used for growth of barley genotypes

**Parameter**	**Unit**	**Soil**	**Sand**
Colour	-	Light brown	Orange
Gravel	%	5-10	5
Texture	-	1	1.5
Ammonium Nitrogen	mg kg^{-1}	1.7	< 1
Nitrate Nitrogen	mg kg^{-1}	8	< 1
Phosphorus (Colwell)	mg kg^{-1}	10	3
Potassium (Colwell)	mg kg^{-1}	135.7	< 15
Sulfur	mg kg^{-1}	6.43	2.5
Organic Carbon	%	0.28	0.10
Conductivity	dS m^{-1}	0.214	< 0.010
pH ($CaCl_2$)	-	7.2	5.3
pH (H_2O)	-	8	6.1
DTPA Copper	mg kg^{-1}	0.32	-
DTPA Iron	mg kg^{-1}	2.46	-
DTPA Manganese	mg kg^{-1}	4.65	-
DTPA Zinc	mg kg^{-1}	0.18	-
Exchangeable Aluminium	meq 100g^{-1}	0.037	-
Exchangeable Calcium	meq 100g^{-1}	2.72	-
Exchangeable Magnesium	meq 100g^{-1}	0.27	-
Exchangeable Potassium	meq 100g^{-1}	0.31	-
Exchangeable Sodium	meq 100g^{-1}	0.23	-
Boron Hot CaCl2	mg kg^{-1}	0.57	-
Phosphorus Buffer Index	-	21.7	6.4
